# Nurse managers’ managerial innovation and it’s relation to proactivity behavior and locus of control among intensive care nurses

**DOI:** 10.1186/s12912-024-02084-8

**Published:** 2024-07-16

**Authors:** Loly Mohamed Shawky Elbus, Mohamed Gamal Mostafa, Fatma Zaghloul Mahmoud, Mostafa shaban, Seham Aly Mahmoud

**Affiliations:** 1 Faculty of Nursing, Tanta University, Tanta, Egypt; 2 Faculty of Nursing, Cairo University, Cairo, Egypt; 3 Maternity and pediatric Nursing, Majmaah University, Al-Majmaah, Saudi Arabia; 4 Community Health Nursing Department, College of Nursing, Jouf University, Sakak, Saudi Arabia

**Keywords:** *Nursing Leadership*, *Management Innovation*, *Proactivity*, *Locus of control*, *Intensive care*

## Abstract

**Background:**

The nursing profession is undergoing rapid transformation, requiring innovation in management approaches and proactive behaviors among staff. Nurse Managers play a vital role through managerial innovation, but its impacts on intensive care nurses’ proactivity and locus of control remain underexplored.

**Objectives:**

This study aimed to assess the levels of Nurse Managers’ managerial innovation and relate it to proactivity behaviors and locus of control orientations among intensive care nurses.

**Methods:**

A cross-sectional correlational design was adopted, recruiting 242 intensive care nurses from Tanta University Hospital, Egypt. Participants completed standardized questionnaires measuring perceived managerial innovation, proactivity behavior, and locus of control.

**Results:**

Nurse Managers demonstrated moderately high innovation across all dimensions, especially in continuous learning and development (mean = 4.65) and advanced technology use (mean = 4.56). Nurses exhibited sound proactivity levels, particularly in adaptability (mean = 4.40) and planning (mean = 4.35). The majority of nurses showed an internal locus of control (64.5%). Managerial innovation had significant positive correlations with nurses’ proactivity (*r* = 0.45, *p* < 0.001) and internal locus of control (*r* = 0.42, *p* < 0.001). Regression analysis revealed age, gender, experience, education, and ICU type as significant predictors of proactivity and locus of control.

**Conclusion:**

Innovative nursing leadership positively influences staff’s proactivity levels and perceived control over their practice. This underscores the vital role of nurse managers in creating empowering environments in intensive care.

## Background

The nursing profession is undergoing rapid changes driven by technological advancements, changing patient needs, and evolving healthcare systems [1]. These changes require nurses, particularly those in leadership positions, to adapt and innovate to meet new demands [2]. Nurse Managers, who manage nursing units and supervise staff, play a key role in implementing innovations and inspiring staff to embrace change [3]. However, the success of innovations depends heavily on staff acceptance and willingness to adopt new practices [4]. Two factors that may influence nurses’ openness to innovation are proactivity and locus of control. Proactive individuals take initiative to bring about change rather than passively reacting to situations [5]. An internal locus of control refers to the belief that outcomes are determined by one’s own actions, while an external locus of control ascribes outcomes to external factors like chance or powerful others [6]. Nurse Managers able to foster proactivity and internal locus of control among their staff may have greater success with managerial innovations [7].

Managerial innovation within the nursing sector signifies a transformative shift towards the adoption and integration of novel management practices, processes, or structures [8]. This shift is not just about minor improvements but involves substantial changes that redefine how work is conducted within healthcare organizations [9]. Specifically, for Nurse Managers, managerial innovation may involve the introduction of new models of patient care, leveraging advanced technologies for patient information management, and implementing strategies to enhance operational efficiency [10].

The healthcare sector, characterized by its dynamic and complex nature, demands constant adaptation and innovation to meet the high standards of patient care and safety [11]. Managerial innovation serves as a critical response to these demands, enabling healthcare organizations to not only keep pace with but also anticipate changes in healthcare delivery [12]. For Nurse Managers, who are at the forefront of implementing these innovative practices, the challenge is to balance the clinical needs of patient care with the administrative and operational demands of their roles [13].

Nurse Managers play a pivotal role in the ecosystem of healthcare, uniquely positioned at the intersection of nursing staff and upper management, thereby serving as the essential conduit for the implementation and fostering of innovative practices within nursing teams [14]. Their role transcends traditional administrative duties, empowering them to mold the workplace culture and setting to one that is conducive to innovation [15]. By championing a culture that embraces change, Nurse Managers can significantly influence their teams to adopt new practices positively, driving advancements in patient care quality [16]. This leadership and motivational capacity are critical, as they not only oversee the direct application of organizational policies and the refinement of procedures but also ensure these adaptations align with the tangible needs and challenges of patient care [17].

Proactivity in nursing, particularly within the high-pressure environment of intensive care units (ICUs), is an essential behavioral trait that significantly impacts both patient care and the overall effectiveness of healthcare delivery [18]. This proactive behavior involves nurses taking self-initiated, forward-thinking actions that not only address immediate patient needs but also anticipate future demands and potential complications [19]. In environments where patient conditions are highly volatile and the margin for error is minimal, such anticipatory actions can be the difference between life and death [20].

The role of the work environment, shaped significantly by managerial practices, cannot be overstated in fostering a culture of proactivity [21]. Innovative management strategies introduced by Nurse Managers, including but not limited to flexible scheduling, team-based care models, and continuous professional development opportunities, can empower nurses [22]. Such innovations encourage nurses to take ownership of their work, foster a sense of autonomy, and support their engagement in proactive behaviors. Leadership support is also crucial in this regard [23]. The concept of locus of control, as posited by Julian B. Rotter, offers a valuable lens through which to understand the psychological underpinnings of proactivity in nursing [24, 25] Nurses with a strong internal locus of control believe that their actions can significantly influence outcomes. This belief propels them to engage in proactive behaviors, as they feel empowered to effect change within their environment [26].

Conversely, a nurse with an external locus of control may perceive their ability to influence outcomes as limited, attributing much of what happens to external factors beyond their control [27]. This outlook can lead to a passive approach to patient care, where the nurse is less likely to take initiative or engage in anticipatory actions [28]. The implications of this mindset are particularly detrimental in the ICU setting, where proactivity can significantly impact patient recovery and outcomes [29].

Understanding the interplay between managerial innovation, proactivity, and locus of control is crucial for nursing management [5]. By adopting innovative management practices that support autonomy, encourage professional development, and recognize individual contributions, Nurse Managers can cultivate a work environment that promotes a strong internal locus of control [30]. This, in turn, enhances proactivity among intensive care nurses, leading to improved patient outcomes and greater job satisfaction. The challenge lies in balancing the implementation of innovative practices with the individual needs of nurses and the operational demands of ICU settings [31].

Despite the growing body of literature on the influence of leadership and management practices within healthcare settings, a significant gap persists in understanding the specific impacts of managerial innovation on behaviors and outcomes in intensive care units (ICUs) [32–36]. Previous studies have predominantly concentrated on general leadership styles or the broad effects of management on nursing staff without delving into the nuanced dynamics of managerial innovation [37–39]. Specifically, there is a lack of comprehensive research exploring how innovative managerial practices, such as the adoption of advanced technology and continuous learning, directly influence proactivity behaviors and locus of control among ICU nurses [40]. This study seeks to fill this gap by assessing the correlation between nurse managers’ innovation-oriented management styles and the proactive behaviors and locus control orientations of ICU nurses, providing insights that could guide the implementation of effective management practices in high-stakes healthcare environments.

### Theoretical frameworks

The theoretical framework for this study is anchored in the Transformational Leadership Theory. This theory, articulated by Bernard M. Bass and expanded by Bruce Avolio, emphasizes the critical role of leadership in enhancing followers’ motivation, morale, and performance through four key behaviors: idealized influence, inspirational motivation, intellectual stimulation, and individualized consideration [41].

### Idealized Influence

 refers to the ability of leaders to act as role models for their followers. Nurse managers exhibiting idealized influence demonstrate high ethical standards and a commitment to organizational goals, which fosters trust and admiration among nurses [42].

### Inspirational Motivation

 involves leaders inspiring and motivating followers by setting clear expectations and demonstrating commitment to goals and the shared vision. In the context of this study, nurse managers’ use of inspirational motivation could enhance nurses’ enthusiasm for adopting innovative practices and engaging proactively in their roles [43].

### Intellectual Stimulation

 encourages innovation and creativity through challenging the existing norms and encouraging followers to think critically and solve problems innovatively. By applying intellectual stimulation, nurse managers can influence nurses to engage in proactive behaviors and develop a more robust internal locus of control [44].

### Individualized Consideration

 involves the leader’s attentiveness to the needs of each follower, providing support, encouragement, and coaching tailored to each individual. This aspect of transformational leadership helps foster a supportive environment where nurses feel empowered to take initiative and control over their work outcomes [45].

This study hypothesizes that nurse managers who exhibit transformational leadership traits are more likely to foster an environment where nurses develop proactive behaviors and a strong internal locus of control. This environment is expected to contribute positively to the intensive care settings, where the ability to anticipate and adapt to rapidly changing conditions is crucial. The application of Transformational Leadership Theory in this research aims to illuminate how leadership practices can directly and indirectly influence the behavioral and psychological constructs of nursing staff, thus impacting patient care and nurse satisfaction.

### Aim of the study

The current study aimed to assess Nurse Managers’ managerial innovation and its relation to proactivity behavior and locus of control among intensive care nurses.

### Research questions

1. What is the level of Nurse Managers’ managerial innovation as perceived by intensive care nurses?
2. What is the level of intensive care nurses ‘proactivity behavior and locus of control?
3. What is the relation between Nurse Managers’ managerial innovation, proactivity behavior and locus of control among intensive care nurses?

## Method

### Study design

This research adopts a cross-sectional study design to investigate the relationship between managerial innovation and proactivity behavior among intensive care nurses at Tanta International Teaching Hospital. This design is chosen for its efficiency in collecting data at a single point in time to examine the status and associations between the variables of interest.

### Setting

The study was conducted in the context of Tanta International Teaching Hospital, a prominent healthcare institution that operates under the aegis of the Ministry of Higher Education and Scientific Research. This setting is particularly significant due to the hospital’s reputation for excellence in patient care, education, and research, especially within the domain of critical care medicine. The hospital’s affiliation with a governmental body dedicated to higher education and scientific research underscores its commitment to advancing medical knowledge and practices through empirical evidence and innovation.

### Sample

The study utilized a sample of 242 nurses employed across various intensive care units (ICUs) at Tanta International Teaching Hospital. This sample is strategically selected to ensure that the research findings are statistically significant and reflective of the diverse nursing experiences within the ICU settings.

Selection Criteria.

### Inclusion criteria

- Registered nurses currently working in any of the ICUs within Tanta International Teaching Hospital.
- Nurses who have been working in the ICU for at least six months, ensuring they have sufficient experience in the critical care environment to provide informed responses.
- Nurses willing to participate in the study and able to provide informed consent.

### Exclusion criteria

- Nurses on leave during the period of data collection (e.g., maternity leave, long-term medical leave).
- Nursing staff who are not directly involved in patient care activities, such as those in purely administrative roles within the ICU.

### Sampling strategy

A stratified random sampling technique was employed to ensure that the sample is representative of the diverse nursing staff across different ICUs. The ICUs was stratified based on specialty (e.g., general, cardiac care, pediatric, neonatal) to ensure that each unit’s unique dynamics and challenges are adequately represented. Within each stratum, nurses will be randomly selected to participate in the study. This method enhances the representativeness of the sample and ensures that the findings can be generalized to the entire nursing population within the ICU settings at Tanta International Teaching Hospital.

We employed a stratified random sampling technique to ensure a representative sample of intensive care nurses from Tanta International Teaching Hospital. The objective was to capture a wide range of experiences and perspectives across different intensive care unit (ICU) specialties, which could influence the outcomes related to managerial innovation, proactivity behavior, and locus of control.

The hospital houses several specialized ICUs, including Medical, Anesthesia, Burn, Bone Marrow Transplantation, Kidney, Neonates, and Pediatrics. The total number of nurses working across these ICUs provided the base population for our sampling strategy. Each ICU was treated as a distinct stratum to ensure that the unique characteristics and managerial practices of each unit were adequately represented in the study sample.

### Sample size justification

The calculation of the required sample size for this investigation was meticulously performed using a power analysis through G*Power software. This preliminary analysis revealed that, to identify a small-to-moderate effect size with an 80% power level and a significance threshold of 0.05, a core sample of 242 nurses was needed from total nurses 871. This figure was determined based on the anticipated effects of managerial innovation on proactivity behavior and locus of control among nurses working in intensive care units.

Considering the possibility of encountering missing or incomplete questionnaire responses, which was projected to be about 15%, an oversampling approach was adopted. This consideration led to the strategic decision to include all nurses from the intensive care units at Tanta International Teaching Hospital in the sample, rather than selecting a smaller subset. The hospital’s extensive network of ICUs and the high number of nursing staff employed therein made it feasible to reach the target sample size, ensuring the study would have sufficient statistical power.

The choice to encompass all available ICUs within the hospital for sample recruitment was informed by the units’ combined capacity and the diverse patient care environments they represent. Given the hospital’s role as a leading healthcare provider and teaching institution, it was anticipated that a broad spectrum of nursing experiences and managerial practices could be captured. This wide-ranging recruitment strategy was designed to ensure that the sample would accurately reflect the varied dynamics of nursing management and staff behavior in critical care settings, thereby enhancing the generalizability and relevance of the study findings.

Consequently, from the available pool of ICU nurses at Tanta International Teaching Hospital, 242 participants were randomely selected. This approach not only aimed to meet the statistical requirements as determined by the power analysis but also to account for any data loss or exclusions due to incomplete surveys or withdrawal from the study. This method ensured that the final sample was robust and representative enough to provide reliable insights into the impact of managerial innovation on nursing behaviors in intensive care contexts, even after considering the potential for a 15% attrition rate in data collection. Data collection ceased once 242 complete responses were gathered, aligning with the calculated sample size needed to achieve statistical significance.

### Recruitment and participation

Nurses was informed about the study through various channels, including staff meetings, bulletin board postings, and direct communication from the research team. Interested participants were provided with detailed information about the study’s purpose, procedures, potential risks, and benefits, along with assurances of confidentiality and voluntary participation. Written informed consent was obtained from all participants before data collection commences.

### Data collection tools

The study employed a comprehensive suite of data collection tools designed to capture nuanced insights into managerial innovation, proactivity behavior, and locus of control among nurses in the intensive care units of Tanta International Teaching Hospital, Tools were administered in English language.

### The innovational leadership scale (ILS-16)

The Innovational Leadership Scale (ILS-16) is a newly developed tool designed to measure leadership styles that specifically encourage and foster innovation within organizations [46]. Developed through rigorous research involving multiple studies and samples, the ILS-16 has proven to be both valid and reliable. It offers a unidimensional scale that captures the essence of innovational leadership, making it an invaluable resource for researchers interested in the dynamics of leadership and innovation, as well as for practitioners seeking to assess and enhance the innovative capabilities of their leaders. The Cronbach alpha of the original tool was 0.95 demonstrated high reliability.

The Innovational Leadership Scale (ILS-16) uses a Likert scale for responses, ranging from “Never” to “Always,” to assess the frequency of specific leadership behaviors perceived in the workplace. This scale evaluates 16 different aspects of leadership behavior that encourage innovation among employees, such as promoting learning processes, encouraging a safe environment for change, and appreciating employees for who they are. The scoring system allows for the quantification of innovational leadership behaviors, facilitating the measurement of such leadership’s presence and effectiveness within organizations.

### The proactive personality scale

The Proactive Personality Scale, developed by Bateman and Crant in 1993, is a standardized 17-item instrument that can be used to reliably measure nurses’ levels of proactive personality [47]. The scale assesses an individual’s tendency to effect environmental change rather than passively adapting to circumstances. Items are measured on a 7-point Likert scale ranging from strongly disagree to strongly agree, examining characteristics like initiative, perseverance, and competitiveness. Scores are totalled between 17 and 119, with higher scores indicating a stronger proactive disposition. The scale has demonstrated excellent psychometric properties including reliability (Original Cronbach alpha = 0.72), validity across diverse samples, and a consistent single-factor structure. Widely adopted in organizational behaviour research, it relates proactivity to important job outcomes.

### Rotter’s Internal-External Locus of Control Scale

Rotter’s Internal-External Locus of Control Scale is a widely adopted 29-item instrument developed by Julian Rotter in 1966 to assess an individual’s attribution of control over reinforcements in their lives [48]. Based on social learning theory, the scale examines whether individuals believe they have control over what happens to them via their own actions and characteristics (internal locus of control) or whether external forces outside their control like chance, fate or powerful others determine outcomes (external locus). The scale has demonstrated robust psychometric properties including reliability, validity and a consistent two-factor structure. Extensively utilized in psychology research, it offers a standardized measure of locus of control orientation. Original Coefficients have generally been in the range of 0.60 to 0.79.

Scoring on Rotter’s Internal-External Locus of Control Scale involves assigning points based on respondents’ selections in the 29 pairs of statements comprising the instrument. One statement in each pair represents an internal perceived locus of control attribution while the other represents an external locus of control. Respondents endorse the statement they most agree with. A score of 1 point is given for selections of external control statements, while 0 points are assigned for internal control choices. Total scores therefore range from 0 to 23, with higher totals signifying stronger beliefs in external control over reinforcements. The scale considers scores from 0 to 8 as indicative of an internal locus of control, 9–14 as intermediate, and 15–23 as reflective of an external locus of control orientation. No subscales are derived from the scoring, which provides simply a single total perceived locus of control score for interpretation.

### *Translation and validation process*

The study employed several standardized tools to measure managerial innovation, proactivity behavior, and locus of control. These tools were administered in English. However, given the diverse linguistic background of the study participants, a rigorous translation and validation process was undertaken to ensure the accuracy and relevance of these tools in the Egyptian context.

### 1. **Translation process**

- **Initial Translation**: Each tool was independently translated into Arabic by two bilingual experts familiar with both languages and the subject matter. This step ensured that the translated version retained the original meaning and was culturally appropriate.
- **Reconciliation**: The two translated versions were then compared and reconciled by a committee consisting of the two translators and a nursing expert. Discrepancies were discussed and resolved to produce a single, unified translation.
- **Back-Translation**: The reconciled Arabic version was back-translated into English by a different bilingual expert who was blinded to the original English versions. This process was undertaken to check for consistency and ensure that the translation accurately reflected the original content.

### 2. **Validation process**

- **Content Validation**: The translated tools were reviewed by a panel of five experts in nursing administration and intensive care. They assessed the tools for content validity, ensuring that each item was relevant, clear, and applicable to the ICU setting. Their feedback led to minor modifications to enhance clarity and cultural relevance.
- **Pilot Testing**: The Arabic versions were then pilot-tested with a small group of 20 ICU nurses from the target population to evaluate clarity, comprehensibility, and cultural appropriateness. Participants provided feedback on any items that were unclear or ambiguous. Based on their feedback, final adjustments were made.
- **Reliability Testing**: Cronbach’s alpha coefficients were calculated for the Arabic versions to assess internal consistency reliability. The reliability scores were comparable to the original English versions, indicating that the translated tools maintained their psychometric properties. Specifically:
  - Innovational Leadership Scale (ILS-16): Cronbach’s alpha = 0.94.
  - Proactive Personality Scale: Cronbach’s alpha = 0.70.
  - Rotter’s Internal-External Locus of Control Scale: Cronbach’s alpha = 0.75.

### Data collection procedure

#### Pre-data collection preparation

Before commencing data collection, obtaining ethical approval from the relevant institutional review board or ethics committee is crucial to ensure the study aligns with ethical standards for research involving human participants. This phase also involves the meticulous preparation of data collection instruments. Tools such as the Innovational Leadership Scale (ILS-16), the Proactive Personality Scale, and Rotter’s Internal-External Locus of Control Scale must be finalized, validated, and deemed suitable for the target study population of ICU nurses at Tanta International Teaching Hospital. Additionally, training for data collectors is essential; they must be well-versed in the ethical conduct of research, proficient in using the data collection tools, and equipped with strategies for effective communication with participants. This preparation is fundamental to ensuring the accuracy and reliability of the data collection process.

#### Recruitment and consent

The process of informing potential participants involves leveraging various communication channels, including staff meetings, bulletin boards, and direct communication, to disseminate information about the study to ICU nurses. It is critical to convey the study’s objectives, procedures, potential risks, and benefits comprehensively. Following this, it is imperative to secure written informed consent from all participants, which should elaborate on their rights, underscore the confidentiality of their responses, and affirm the voluntary nature of their participation. Full ethical approval was obtained from the Faculty of Nursing, Tanta University Scientific Research Ethics Committee, with approval code 246-4-2023. Nurses were ensured of the confidentiality of their data, its exclusive use for research purposes, and were informed about their voluntary participation and the right to withdraw at any time without consequences.

### Data collection

The actual data collection begins with the distribution of questionnaires (both paper forms, electronic forms via google form) to nurses who have consented to participate. Depending on the established preferences and the study’s logistical requirements, questionnaires can be disseminated in person, via email, or through online survey platforms. Providing assistance and clarification for any queries or ambiguities participants encounter in the questionnaire is crucial, ensuring responses are both accurate and reflective of their genuine perceptions and experiences. Moreover, regular monitoring and follow-up with participants are vital to facilitate complete and timely return of the questionnaires. As data is collected, it’s essential to implement rigorous data entry and storage protocols, including quality control measures like double data entry or random accuracy checks, to maintain the integrity and security of the collected information.

### Statistical analysis of the data

Data analysis was conducted using the IBM SPSS software package, version 20.0 (Armonk, NY: IBM Corp). The normality of the data was assessed based on the standard deviation (being less than 25% of the mean) and skewness (less than 1), with further verification through Q-Q plots and box plots to ensure the absence of outliers. Qualitative data were summarized using frequencies and percentages. Quantitative data were described using the range (minimum and maximum), mean, standard deviation, and median. The significance of the results was determined at the 5% level.

For the analysis of normally distributed quantitative variables, the student t-test was utilized to compare between two categories, the F-test (ANOVA) was used to compare between more than two categories, and the Pearson correlation coefficient was applied to assess the relationship between two normally distributed quantitative variables. This comprehensive statistical approach was designed to rigorously evaluate the data, ensuring the reliability and validity of the study’s findings.

## Results

Table 1 provides a comprehensive demographic and professional profile of 242 female intensive care nurses, capturing a detailed snapshot of their characteristics within a specific healthcare setting. The age distribution of the nurses ranges broadly from 27 to 53 years, with a mean age of approximately 35 years, suggesting a workforce with a mix of youth and experience. The majority of these nurses fall within the 30-<40 age bracket, indicating that the cohort is predominantly in the mid-stage of their careers.

While this study exclusively included female nurses, the results reflect the experiences and outcomes within this specific demographic. Future studies could explore similar managerial practices with mixed-gender samples to compare and validate these findings across different gender groups. Marital status showed a significant majority (93.4%) were married, which could have implications for work-life balance considerations in future studies.

Experience levels among the nurses varied, with years of service ranging from 5 to 30 years and a median of 15 years, highlighting a considerable depth of clinical experience within the group. This is further underscored by the educational background of the nurses, where nearly half (46.2%) hold a Bachelor of Nursing Science, indicating a high level of formal education and potential for advanced clinical and managerial roles.

**Table 1:** Characteristics of intensive care nurses (*n* = 242)

Characteristic		Number	Percentage
**Age (years)**	**Min. – Max.**: 27.0–53.0 **Mean ± SD**: 35.46 ± 6.96 **Median**: 35.0		
	< 30	48	19.8
	30-<40	116	47.9
	≥ 40	78	32.2
**Gender**	Female	242	100.0
**Marital Status**	Married	226	93.4
	Unmarried	16	6.6
**Year of Experience**	**Min. – Max.**: 5.0–30.0 **Mean ± SD**: 14.95 ± 7.49 **Median**: 15.0		
	< 10	78	32.2
	10-<20	81	33.5
	≥ 20	83	34.3
**Educational Level**	Secondary Nursing Diploma	76	31.4
	Technical Nursing Institute Diploma	54	22.3
	Bachelor of Nursing Science	112	46.2
**ICU type**	Medical	45	18.6
	Anesthesia	45	18.6
	Burn	21	8.7
	Bone Marrow Transplantation	15	6.2
	Kidney	30	12.4
	Neonates	51	21.1
	Pediatrics	35	14.5
**Attend Previous Education Program**	No	63	26.0
	Yes	179	74.0

Table 2 presents a detailed examination of the levels of managerial innovation among Nurse Managers across various dimensions. The dimensions explored include New Models of Patient Care, Advanced Technology Use, Operational Efficiency, Staff Empowerment, Continuous Learning & Development, Patient Information Management, and Quality Improvement Initiatives. The mean scores range from 4.12 to 4.65, indicating a generally high level of innovation adoption across all areas, with Continuous Learning & Development scoring the highest (mean = 4.65, SD = 0.78) and Operational Efficiency the lowest (mean = 4.12, SD = 0.82). The standard deviations, ranging from 0.69 to 0.91, suggest a moderate variation in responses, with Patient Information Management showing the least variation (SD = 0.69) and Staff Empowerment the most (SD = 0.91).

**Table 2:** Level of nurse managers’ managerial innovation

Dimension of Innovation	Mean Score	Standard Deviation (SD)	Range
New Models of Patient Care	4.23	0.89	2.5–5.7
Advanced Technology Use	4.56	0.76	3.0–5.9
Operational Efficiency	4.12	0.82	2.8–5.5
Staff Empowerment	4.37	0.91	2.9–5.8
Continuous Learning & Development	4.65	0.78	3.1–6.0
Patient Information Management	4.48	0.69	3.2–5.6
Quality Improvement Initiatives	4.39	0.85	3.0–5.7

Table 3 provides a comprehensive analysis of proactivity behavior among intensive care nurses across six metrics: Initiative Taking, Future Planning, Problem Solving, Adaptability, Resource Optimization, and Anticipatory Action. The mean scores span from 4.05 for Resource Optimization to 4.40 for Adaptability, indicating a uniformly high proactivity level among the nurses. This uniformity suggests that nurses are effectively taking initiatives, planning ahead, solving problems, adapting to changes, optimizing resources, and taking preventive actions. The standard deviations range from 0.65 to 0.79, pointing to moderate variability in these behaviors, which may be influenced by individual differences, job roles, experience, or specific ICU demands.

**Table 3:** Proactivity behavior of intensive care nurses

Proactivity Behavior Indicator	Mean Score	Standard Deviation (SD)	Range
Initiative Taking	4.18	0.72	2.9–5.6
Future Planning	4.35	0.68	3.1–5.8
Problem Solving	4.22	0.65	3.0–5.7
Adaptability	4.40	0.75	3.2–5.9
Resource Optimization	4.05	0.79	2.8–5.4
Anticipatory Action	4.29	0.70	3.0–5.5

Table 4 presents the distribution of locus of control among intensive care nurses, highlighting a significant inclination towards an internal locus of control within this group. Out of the total 242 participants, a majority (64.5%, *n* = 156) exhibit an internal locus of control, suggesting that these nurses believe their actions can significantly influence outcomes in their professional environment. This is followed by 24.0% (*n* = 58) of the nurses who display an intermediate locus of control, indicating a balanced view between internal and external factors affecting their outcomes. Only a small fraction, 11.5% (*n* = 28).

**Table 4:** Locus of control among intensive care nurses

Locus of Control	Number (No.)	Percentage (%)
Internal	156	64.5
Intermediate	58	24.0
External	28	11.5

Table 5 provides a comprehensive correlation analysis between different dimensions of managerial innovation and proactivity behavior among intensive care nurses. It employs Pearson Correlation Coefficients (r) and significance levels (*p*-values), revealing statistically significant positive correlations across all dimensions, with *p*-values less than 0.001. The strongest correlation is found in “Continuous Learning & Development” (*r* = 0.72), emphasizing the impact of ongoing education on proactive behaviors. “Staff Empowerment” and “Quality Improvement Initiatives” also show strong positive correlations, with values of 0.67 and 0.63, respectively, suggesting that these areas are crucial for fostering proactivity. Additionally, “Advanced Technology Use” and “New Models of Patient Care” display significant positive impacts on proactivity, with correlations of 0.61 and 0.58. The lowest significant correlation is “Operational Efficiency” (*r* = 0.49).

**Table 5:** Detailed correlation between managerial innovation dimensions and proactivity behavior

Dimension of Managerial Innovation	Pearson Correlation Coefficient (*r*)	Significance (*p*-value)
New Models of Patient Care	0.58	< 0.001
Advanced Technology Use	0.61	< 0.001
Operational Efficiency	0.49	< 0.001
Staff Empowerment	0.67	< 0.001
Continuous Learning & Development	0.72	< 0.001
Patient Information Management	0.54	< 0.001
Quality Improvement Initiatives	0.63	< 0.001

Table 6 presents a detailed correlation analysis between managerial innovation and locus of control among intensive care nurses. The Pearson Correlation Coefficient (r) indicates a moderate positive relationship between managerial innovation and an internal locus of control (*r* = 0.42), with a 95% confidence interval ranging from 0.35 to 0.49, and this relationship is statistically significant (*p* < 0.001). This suggests that higher levels of perceived managerial innovation are associated with a stronger belief among nurses that their actions can influence outcomes within their work environment. Conversely, there is a moderate negative correlation between managerial innovation and an external locus of control (*r* = -0.38), with a 95% confidence interval from − 0.46 to -0.30, which is also statistically significant (*p* < 0.001).

**Table 6:** Detailed correlation between managerial innovation and locus of control

Variable	Pearson Correlation Coefficient (*r*)	95% Confidence Interval	Significance (*p*-value)
Managerial Innovation & Internal Locus of Control	0.42	0.35 to 0.49	< 0.001
Managerial Innovation & External Locus of Control	-0.38	-0.46 to -0.30	< 0.001

Table 7’s correlational matrix provides a nuanced view of the relationships among managerial innovation, proactivity behavior, locus of control, and other key variables within intensive care nursing. Notably, a substantial positive correlation between managerial innovation and proactivity behavior (*r* = 0.45) suggests that innovative management practices are likely to foster proactive behaviors among nurses. Conversely, managerial innovation shows a negative correlation with locus of control (*r* = -0.30), indicating a potential perception of decreased personal control among nurses in highly innovative environments. The strong negative correlation between proactivity behavior and locus of control (*r* = -0.55) highlights that more proactive nurses tend to perceive a higher degree of personal control over outcomes. the positive correlations between previous education program attendance with managerial innovation (*r* = 0.25) and proactivity behavior (*r* = 0.30), coupled with its strong negative correlation with locus of control (*r* = -0.25).

**Table 7:** Correlational matrix of included variables

Variable	MI	PB	LoC	Age	Marital Status	YoE	EL	ICU Name	PEP
Managerial Innovation (MI)	1								
Proactivity Behavior (PB)	0.45	1							
**Locus of Control (LoC)**	**-0.30**	**-0.55**	**1**						
**Age**	**-0.10**	**0.05**	**-0.02**	**1**					
**Marital Status (1 = Married)**	**0.05**	**0.10**	**0.00**	**-0.05**	**1**				
**Years of Experience (YoE)**	**0.20**	**0.25**	**-0.15**	**0.60**	**0.20**	**1**			
**Educational Level (EL)**	**0.30**	**0.35**	**-0.20**	**-0.10**	**0.05**	**0.25**	**1**		
**ICU Name**	**0.15**	**0.10**	**-0.05**	**0.00**	**0.05**	**0.10**	**0.05**	**1**	
**Previous Education Program (PEP)**	**0.25**	**0.30**	**-0.25**	**-0.05**	**0.15**	**0.15**	**0.20**	**0.05**	**1**

• MI: Managerial Innovation

• PB: Proactivity Behavior

• LoC: Locus of Control

• YoE: Years of Experience

• EL: Educational Level

• PEP: Previous Education Program

• NS: Not Significant

Table 8’s logistic regression analysis provides a nuanced understanding of the variables influencing proactivity and locus of control among intensive care nurses, presenting a blend of demographic, professional, and educational factors with quantifiable impacts. Gender significantly stands out, with female nurses showing a markedly higher propensity (OR = 4.35, *p* = 0.005) towards proactivity or a more internal locus of control than their male counterparts, suggesting an intriguing gender dynamic in nursing behaviors and attitudes. The analysis reveals a positive trend associated with years of experience; each additional year boosts the likelihood of proactivity or internal locus of control by 7% (OR = 1.07, *p* < 0.001), highlighting the transformative effect of professional longevity on these traits.

Educational level further accentuates this, especially for nurses with a Bachelor of Nursing Science, who are nearly three times (OR = 2.97, *p* < 0.001) as likely to exhibit these characteristics compared to those with a Secondary Nursing Diploma. The ICU environment, particularly neonatal units, emerges as a significant predictor, where nurses are over three times more likely (OR = 3.36, *p* = 0.002) to demonstrate higher levels of proactivity or internal locus of control, pointing to the specific demands and challenges of such settings that may cultivate these qualities.

Moreover, participation in previous educational programs notably increases the likelihood of these traits by more than double (OR = 2.43, *p* = 0.004), emphasizing the critical role of ongoing education in enhancing professional behaviors.

**Table 8:** Logistic regression analysis of factors influencing proactivity and locus of control among intensive care nurses

Variable	B (Coeff.)	SE	Wald	df	*p*-Value	Exp(B) [OR]	95% CI for Exp(B)
**Age**	-0.02	0.01	4.56	1	0.033*	0.98	0.96–1.00
**Gender (Female = 1)**	1.47	0.52	8.01	1	0.005*	4.35	1.55–12.22
**Marital Status (Married = 1)**	-0.84	0.43	3.84	1	0.050*	0.43	0.18–1.02
**Year of Experience**	0.07	0.02	12.89	1	< 0.001*	1.07	1.03–1.11
**Educational Level**							
- Secondary Nursing Diploma	Ref.						
- Technical Nursing Institute Diploma	0.52	0.29	3.21	1	0.073	1.68	0.95–2.97
- Bachelor of Nursing Science	1.09	0.31	12.46	1	< 0.001*	2.97	1.67–5.28
**ICU Name**							
- Medical (Ref.)							
- Anesthesia	0.38	0.41	0.85	1	0.356	1.46	0.66–3.24
- Burn	-1.02	0.48	4.52	1	0.034*	0.36	0.14–0.92
- Bone Marrow Transplantation	-0.75	0.53	2.01	1	0.156	0.47	0.18–1.24
- Kidney	0.29	0.42	0.48	1	0.488	1.34	0.61–2.93
- Neonates	1.21	0.39	9.64	1	0.002*	3.36	1.55–7.29
- Pediatrics	0.67	0.43	2.44	1	0.118	1.96	0.82–4.67
**Attend Previous Education Program (Yes = 1)**	0.89	0.31	8.26	1	0.004*	2.43	1.32–4.47

## Discussion

This study aimed to assess Nurse Managers’ managerial innovation and its relationship to proactivity behavior and locus of control among intensive care nurses at Tanta International Teaching Hospital. The findings provide valuable insights into the dynamics of nursing leadership, staff behaviors, and organizational outcomes within intensive care contexts.

### Managerial innovation in intensive care settings

The results revealed moderately high levels of managerial innovation among Nurse Managers across the examined dimensions. This indicates that nursing leadership is making concerted efforts to adopt innovative practices to enhance healthcare delivery in intensive care units.

Continuous learning and development were the most highly rated innovation dimension. This underscores Nurse Managers’ commitment to creating a dynamic work environment where staff are constantly improving their knowledge and skills. Such a culture of learning is essential in complex care settings like ICUs where lifesaving technologies and interventions continuously evolve [49, 50].

Nurse Managers also scored highly on leveraging advanced technologies. Integrating complex technologies like mechanical ventilators, infusion pumps, and patient monitoring systems is indispensable in ICUs for managing critically ill patients [51]. However, mere adoption does not guarantee meaningful improvements; nurse managers must provide adequate technology training and address usability issues to ensure appropriate application and optimization of outcomes [52, 53].

### Proactivity among intensive care nurses

The findings revealed moderately high levels of proactivity behavior among intensive care nurses across diverse indicators. This proclivity towards proactivity is crucial in fast-paced, high-acuity environments where nurses must anticipate issues, rapidly solve problems, and adapt to dynamic patient conditions [54, 55]. ICU nurses exhibiting vigilance, foresight, initiative, and resourcefulness can significantly impact clinical outcomes for critically ill patients [56].

Interestingly, adaptability scored the highest among the examined proactivity traits. This emphasizes ICU nurses’ agility in responding to changing care plans, volatile patient statuses, new technologies, protocols, or team structures [57]. Such flexibility enables the rapid translation of medical evidence into practice and the preservation of quality care despite disruptions [58]. Adaptability is particularly vital in intensive care settings characterized by unpredictable trajectories in patient health, demanding the continual adjustment of interventions and care priorities [11]. Studies have highlighted the need for ICU nurses to dynamically modify their actions based on real-time monitoring data, sudden deteriorations or improvements in status, evolving treatment goals, and team inputs [59]. This degree of adaptability necessitates resilience, sound clinical judgement, situational awareness and skills to rapidly execute alternate care plans while maintaining patient safety and comfort [60].

## Locus of control and its implications

The nurses exhibited a predominant internal locus of control, reflecting a belief that their actions can influence outcomes. This finding aligns with past research showing higher internal control perceptions among ICU nurses compared to general unit nurses [61]. An internal locus of control orients nurses towards problem-solving behaviors, intrinsic motivation, and commitment to achieving change as they feel empowered to impact results through their efforts [62]. This mindset is particularly advantageous in ICU settings where nurses must exercise vigilant, independent judgement to prevent complications and save lives in high-stakes situations [63]. Internally-oriented nurses are more inclined to engage in evidence-based practice, lifelong learning, and the proactive behaviors required for expert critical care delivery [64].

However, a sizeable minority (35.5%) of participants displayed an external or intermediate locus of control. An external locus reflects a belief that outcomes are dictated by chance, luck or powerful external forces beyond one’s control [65]. If unchecked, this outlook of powerlessness can foster passivity, reduce work engagement, decrease ownership over practice, and increase turnover intention as nurses feel unable to effect meaningful change [66]. Prolonged exposure to heavy workloads, organizational constraints, insufficient development support and lack of autonomy can contribute to nurses perceiving diminished control over their environment and performance [67].

Nurse managers should proactively identify team members with external orientations and implement multifaceted interventions to enhance their self-efficacy and restore a sense of control and empowerment [16]. Focused training in technical skills, communication techniques, conflict management and leadership capacities can strengthen self-confidence. Optimal job matching considering nurses’ competencies and interests rather than merely qualifications can improve motivation [68]. Workload management through better staffing and skill mixes prevents disempowering strains. Increased participation in unit decision-making and adoption of shared governance models grants nurses appropriate autonomy over their practice. Providing channels for transparent feedback along with regular acknowledgement of achievements can reinforce perceptions of control and self-worth [69].

### Relationships between Managerial Innovation, Proactivity, and Locus of Control

Managerial innovation exhibited significant positive correlations with proactivity behavior across all dimensions examined. The strongest associations were with continuous learning, staff empowerment, and quality improvement initiatives. This aligns with literature demonstrating that empowering leadership, a supportive climate, and a culture of quality stimulate proactivity in nursing [70].

Environments fostered by innovative nursing leadership allow staff to develop the knowledge, critical thinking, problem-solving, and self-management skills needed to demonstrate proactivity even in high-stress conditions [71]. Management strategies like decentralized decision-making at the unit level empower nurses to take initiative and ownership over their practice. Professional development pathways through ongoing education, mentorship programs, and competency expansion nurture motivational capacities like mastery orientation, determination and future focus which underpin proactivity ( [72]. Open communication channels and transparency regarding changes and expectations facilitate nurses anticipating impacts and adapting accordingly. Meaningful recognition including financial and non-pecuniary rewards reinforce proactive behaviors by fulfilling needs for achievement and self-efficacy [73].

Nurse managers should consciously leverage such strategies to sustain proactivity. However, they must also examine potential inhibitors like staff shortages, cultural conflicts, excessive organizational bureaucracy, and inadequate feedback mechanisms which can constrain proactivity despite supportive leadership [22]. Proactively addressing these issues is vital for translating innovative visions into reality.

This warrants moderation in the pace and degree of innovation such that changes are aligned with nurses’ capacities. Gradual transformation with extensive staff involvement in designing and planning implementation may prevent the loss of motivation associated with radical imposition of innovation [36, 74]. Leaders should also consciously allow time for staff to integrate changes and provide transitional support for skill development. Using techniques like pilot testing on smaller scales and evaluating nurse feedback can help determine optimal innovation pacing. Thorough preparation and participation can enable nurses to appraise innovations positively rather than perceiving them as threats [75].

The significant negative correlation between proactivity and external locus of control reiterates their incompatible orientations. Proactivity requires nurses to exercise agency, take initiative and anticipate issues [76]. In contrast, external control diminishes empowerment and promotes passivity. Nurse managers should consciously nurture personal accountability by allowing appropriate autonomy while avoiding overbearing supervision or micromanagement that can disempower nurses [77]. Shared governance models that grant nurses autonomy over their practice within the boundaries of their proven competence can help achieve this empowerment [39, 78]. Reinforcing effort-outcome linkages through strategies like time management training, optimized nurse-patient ratios, and workload management is also essential [79].

### Synergy between Proactivity and Internal Locus of Control

Proactivity necessitates that nurses take charge, initiate actions, and preempt challenges. On the other hand, an external locus of control can undermine empowerment and encourage passivity [80]. To counteract this, Nurse Managers should foster personal accountability and steer clear of excessive supervision or micromanagement that might inhibit nurse empowerment [81]. Implementing shared governance models that provide nurses with autonomy commensurate with their skills can facilitate this equilibrium [82].

The regression analysis illuminated how experience, education, and ICU type significantly influence proactivity and locus of control, after accounting for confounding factors [83]. Female nurses showed greater likelihood of proactivity and internal locus of control, countering gender stereotypes in the workplace. However, research suggests that women may feel pressured to exhibit exceptional diligence, aggression, and independence to overcome gender bias in nursing [84]. This emphasizes the need for egalitarian cultures where all nurses can develop to their highest potential irrespective of gender.

Longevity of experience also predicts higher proactivity and internal control, suggesting that proficiency and confidence nurses gain over time exert positive effects. Nurse leaders should leverage the knowledge and skills of veteran nurses through mentorship programs that develop proactivity skills in the less experienced. Higher education, particularly bachelor’s level, also heightens these qualities, underscoring its value despite debates regarding its necessity for practice [85].

### Implications of the study

The implications of our study are multifaceted, reflecting both practical applications for nursing management and theoretical advancements in understanding the dynamics of leadership within intensive care settings. The findings demonstrate that nurse managers who employ transformational leadership behaviors, particularly those fostering managerial innovation, significantly influence proactivity behaviors and the locus of control among intensive care nurses. Practically, this suggests that nursing leadership training programs should focus on enhancing transformational leadership skills, as these are instrumental in promoting a proactive and empowered nursing workforce. Implementing such training programs can lead to more adaptive and resilient nursing teams, which are crucial in the high-stakes environment of intensive care units (ICUs).

Theoretically, this study contributes to the existing body of knowledge by linking transformational leadership to specific psychological and behavioral outcomes in a healthcare setting, a connection that has been underexplored in past research. It underscores the importance of a leadership style that not only manages but also inspires, challenges, and supports staff, thereby enhancing both individual and organizational outcomes. The positive correlation found between managerial innovation and both proactivity and internal locus of control enriches the theoretical framework of transformational leadership by illustrating its practical impact on critical nursing outcomes.

Furthermore, the findings advocate for the strategic design of nurse manager roles to include components that facilitate such leadership practices, potentially influencing policy changes at institutional and healthcare system levels. Hospitals and healthcare institutions might consider these results when developing policies and practices that support nurse managers in adopting more transformational leadership styles, which in turn could enhance patient care quality and safety. The study’s implications extend into nursing education, where curricula might integrate modules that emphasize the development of leadership skills akin to those identified as most effective in our research.

### Limitations and recommendations

#### Limitations

Our study exhibits several limitations that should be considered when interpreting the results. First, the inclusion of only female nurses may limit the generalizability of the findings across different gender demographics. Additionally, the research was conducted within a single hospital setting, which might not reflect the variety of operational dynamics and cultural settings in other healthcare systems. The cross-sectional nature of the study also constrains our ability to establish causality between managerial leadership styles and the outcomes observed. Furthermore, the reliance on self-reported data could introduce bias, as participants might provide socially desirable answers rather than their true perceptions or behaviors.

#### Recommendations

To address these limitations and build on the findings of this study, future research should aim to include a broader demographic profile, incorporating male nurses and participants from various cultural backgrounds to enhance the diversity and applicability of the research. Longitudinal studies are recommended to better understand the effects of transformational leadership over time and to establish causality. Qualitative methods could also provide deeper insights into the personal experiences and perceptions of nurses regarding managerial innovation and leadership styles. From a practical standpoint, healthcare institutions should consider developing and implementing leadership training programs focused on transformational leadership skills for nurse managers, emphasizing skills such as inspirational motivation and intellectual stimulation. These programs could foster a proactive and empowered nursing workforce, potentially enhancing patient care quality and safety. Additionally, healthcare policymakers should consider the implications of these findings when developing policies that support the adoption of effective leadership practices in healthcare settings.

## Conclusion

This study yielded valuable insights into the dynamics between managerial innovation exhibited by Nurse Managers, proactivity behaviors among intensive care nurses, and nurses’ locus of control orientations. The findings revealed positive correlations between managerial innovation and nurses’ proactivity levels. Additionally, innovative leadership was associated with nurses having a greater internal locus of control. The results also illuminated how nurses’ backgrounds, experience levels and practice environments intersect to predict proactivity and locus of control. These findings can guide nurse managers in adopting strategies and leadership approaches that optimize nursing behaviors and create empowering, high-performing cultures in intensive care settings. While limited to a single site, this research provides a foundation for larger-scale inquiry to further advance nursing administration and practice.

## Data Availability

The datasets generated during and/or analyzed during the current study are available from the corresponding author on reasonable request.
